# Acute Effects of Single Versus Combined Inhaled β2-Agonists Salbutamol and Formoterol on Time Trial Performance, Lung Function, Metabolic and Endocrine Variables

**DOI:** 10.1186/s40798-023-00630-3

**Published:** 2023-08-28

**Authors:** Daniel A. Bizjak, Dorle Nussbaumer, Kay Winkert, Gunnar Treff, Kensuke Takabajashi, Lennart Mentz, Franziska Schober, Jasmine-Lèonike Buhl, Lucas John, Jens Dreyhaupt, Luise Steeb, Lukas C. Harps, Maria K. Parr, Patrick Diel, Martina Zügel, Jürgen M. Steinacker

**Affiliations:** 1 Department of Internal Medicine, Division of Sports and Rehabilitation Medicine, University Hospital Ulm, 89075 Ulm, Germany; 2 Institute of Epidemiology and Medical Biometry, Ulm University, 89075 Ulm, Germany; 3 Pharmaceutical Analysis and Metabolism, Institute of Pharmacy, Freie Universität Berlin, 14195 Berlin, Germany; 4 Institute of Cardiovascular Research and Sports Medicine, Molecular and Cellular Sports Medicine, German Sport University Cologne, 50933 Cologne, Germany; 5 Institute of Sports Medicine, Paracelsus Medical University Salzburg, 5020 Salzburg, Austria

**Keywords:** Anti-doping, Performance-enhancing methods, Beta 2 agonists, Muscle metabolism, Detection methods, Sex-specific thresholds

## Abstract

**Background:**

High prevalence rates of β2-agonist use among athletes in competitive sports makes it tempting to speculate that illegitimate use of β2-agonists boosts performance. However, data regarding the potential performance-enhancing effects of inhaled β2-agonists and its underlying molecular basis are scarce.

**Methods:**

In total, 24 competitive endurance athletes (12f/12m) participated in a clinical double-blinded balanced four-way block cross-over trial to investigate single versus combined effects of β2-agonists salbutamol (SAL) and formoterol (FOR), to evaluate the potential performance enhancement of SAL (1200 µg, Cyclocaps, Pb Pharma GmbH), FOR (36 µg, Sandoz, HEXAL AG) and SAL + FOR (1200 µg + 36 µg) compared to placebo (PLA, Gelatine capsules containing lactose monohydrate, Pharmacy of the University Hospital Ulm). Measurements included skeletal muscle gene and protein expression, endocrine regulation, urinary/serum β2-agonist concentrations, cardiac markers, cardiopulmonary and lung function testing and the 10-min time trial (TT) performance on a bicycle ergometer as outcome variables. Blood and urine samples were collected pre-, post-, 3 h post- and 24 h post-TT.

**Results:**

Mean power output during TT was not different between study arms. Treatment effects regarding lung function (*p *< 0.001), echocardiographic (left ventricular end-systolic volume *p *= 0.037; endocardial global longitudinal strain *p *< 0.001) and metabolic variables (e.g. NR4A2 and ATF3 pathway) were observed without any influence on performance. In female athletes, total serum β2-agonist concentrations for SAL and FOR were higher. Microarray muscle gene analysis showed a treatment effect for target genes in energy metabolism with strongest effect by SAL + FOR (NR4A2; *p *= 0.001). Of endocrine variables, follicle-stimulating hormone (3 h Post–Post-TT), luteinizing hormone (3 h Post–Pre-TT) and insulin (Post–Pre-TT) concentrations showed a treatment effect (all *p *< 0.05).

**Conclusions:**

No endurance performance-enhancing effect for SAL, FOR or SAL + FOR within the permitted dosages compared to PLA was found despite an acute effect on lung and cardiac function as well as endocrine and metabolic variables in healthy participants. The impact of combined β2-agonists on performance and sex-specific thresholds on the molecular and cardiac level and their potential long-term performance enhancing or health effects have still to be determined.

*Trial registration*: Registered at Eudra CT with the number: 2015-005598-19 (09.12.2015) and DRKS with number DRKS00010574 (16.11.2021, retrospectively registered).

**Supplementary Information:**

The online version contains supplementary material available at 10.1186/s40798-023-00630-3.

## Key Points

• Combined β2-agonist application in threshold doses according to World Anti-Doping Agency (WADA) standards does not result in acute enhanced high-intensity endurance performance in healthy male and female athletes.

• Sex-specific thresholds have to be considered as female sex showed significantly higher β2-agonist serum concentrations compared to their male counterparts.

• Acute effects on lung function and cardiac variables are observed with presumably no performance-enhancing effects in competitions of short duration, but effects in longer time trials or long-term health effects have to be considered.

**Supplementary Information:**

The online version contains supplementary material available at 10.1186/s40798-023-00630-3.

## Background

High prevalence rates of β2-agonists use particularly among athletes of endurance disciplines, combined with data showing that β2-agonist users among Olympic athletes have consistently outperformed their competitors [1], makes it tempting to speculate that illegitimate use and misuse potential of β2-agonists beyond medical reason might be a common practice in competitive sports. Misuse of β2-agonists has been reported in top level cyclists like Chris Froome (adverse analytical finding twice the permissible limit of salbutamol—allegedly due to unusual excretion due to combined medications because of sickness [2]) or world-class triathlete Lisa Roberts (falsely declared usage of an asthma medication that contained a combined medication of fluticasone and vilanterol [3]). Interestingly, the use of asthma medications—especially the combined use of β2-agonists and inhaled corticosteroids—has increased from 9.4% (2002) to 12.6% (2009) in Finnish Olympic athletes, while the prevalence of physician diagnosed asthma cases in the general population remained unchanged[4]. A more recent study examined the asthma prevalence of Finnish cross-country skiers competing on the national level and a higher prevalence in athletes compared to the general population was observed and, additionally, the prevalence of asthma was the highest in the most successful cross-country skiers [5]. In contrast, regarding the medals won by athletes with a therapeutic use exemption in the Games between 2010 and 2018—without special respect to asthma treatment—are only of minor relevance (^~^ 1% of all medals) [6].

Asthma/airway hyper-responsiveness (AHR) is the most common chronic medical condition among Olympic athletes with the highest prevalence rates found in endurance (25%), aquatic (40%) and winter-based (30%) sporting disciplines, and the prevalence remained stable between 1990 and 2020 [7, 8]. The mean asthma prevalence and the use of asthma medication is up to fourfold higher in athletic populations compared to the general public (5%) [9, 10]. Although the 2023 GINA (Global Initiative For Asthma) guidelines for asthma treatment recommend and prefer the use of inhaled corticosteroids (ICS) instead of short-acting β2-agonists (SABA) [11], using combined ICS and long-acting β2-agonist formoterol (LABA) as first choice of treatment, β2-agonists are still common treatment medications due to their easy application and faster wash-out times [12].

Since the 2022-World Anti-Doping Agency (WADA) list of prohibited substances and methods, use of the SABA salbutamol as well as LABAs vilanterol and formoterol, when taken in therapeutic doses (salbutamol: 600 µg/8 h and 1600 µg/24 h, formoterol: 54 µg/24 h, inhaled vilanterol 25 µg/24 h), are permitted in aerosol form ‘in-competition and out-of-competition’ without a therapeutic use exemption, while other β2-agonists such as reproterol or terbutaline and others are prohibited at all time by WADA [13].

The bronchodilatory effect of β2-agonists by relaxing airway smooth muscle for treatment purposes is their most important application use. But the use of β2-agonists and its high prevalence in competitive sports have led to extensive research regarding the potential performance-enhancing effects besides the therapeutic bronchodilatory application. Recent meta-analyses showed that β2-agonists in non-asthmatics have different performance-enhancing advantages, partly depending on type and time of exercise, dose or duration of treatment [14, 15], underlining the complex and still unresolved issue of endurance or strength enhancement by β2-agonists in healthy individuals.

Newer generation β2-agonists, such as formoterol and salmeterol, have been shown to elicit an anabolic response even at very low doses in rats [16]. In addition to increasing muscle size and strength, β2-agonists have also been observed to affect several aspects of skeletal muscle biology, which play important physiological roles in muscle regeneration and energy balance, hence, contributing to increased physical performance levels (e.g. modulation of oxidative metabolism, triglyceride lipolysis, glucose transport, glycogenolysis, muscle protein turnover and satellite cell activation) [17, 18].

There is an increase in scientific evidence to suggest that significant changes in metabolic and trophic programs at the core of skeletal muscle plasticity are adaptively regulated by adrenergic stimulation, involving adrenergic receptor activation, turnover and downstream signalling via the recently described members of the nuclear hormone receptor (NR) family NR4A subgroup (Nur77, Nurr1 and Nor-1) [19, 20].

Data on dose-dependent ergogenic effects of combined β2-agonists are scarce, whereas there is more evidence on the performance impact of SABAs and LABAs in healthy and athletic individuals. Some studies have found no significant effects of inhaled β2-agonists on aerobic capacity and exercise performance in non-asthmatic athletes [21–23], while others report increased muscle strength, endurance and neuromuscular performance [24–26], depending on the dose and route of administration. Here, more potent effects were observed for oral administration [24], whereas a systemic review and meta-analysis of application per inhalation showed possible positive effects on physical performance in healthy subjects, but the underlying results are basing on weak evidence [27]. Moreover, high doses of inhaled β2 agonists lead to elevated plasma levels of the β2-agonists and thus to systemic effects like increased cardiac output [28].

Combining low doses (micro-dosing) of different drug formulations (cocktail formulations) to achieve additive/synergistic effects while matching the individual detection threshold values is a common doping practice [29–31]. One published study has shown that the combined inhalation of salbutamol, formoterol and salmeterol increases swim ergometer performance and quadriceps maximal voluntary isometric contraction in elite swimmers with and without AHR [32].

Due to methodological limitations of existing studies, it is currently unknown whether or not inhaled β2-agonists enhance performance by stimulatory effects in skeletal and cardiac muscle. In addition, the current literature does not allow conclusions on the potential for misuse and the performance-enhancing effects of inhaled β2-agonists. To this end, the present study investigated single versus combined threshold doses of non-prohibited, short-acting (salbutamol) and long-acting (formoterol) β2-agonists effects in order to evaluate their potential performance-enhancing and health effects with focus on time trial performance (primary endpoint), skeletal muscle expression of nuclear NR4A receptors, endocrine regulation, urinary and plasma β2-agonist concentrations, cardiac biomarkers and cardiopulmonary function (secondary endpoints). These data should contribute for supporting WADA in creating a refined annual list of prohibited substances (WADA 2022), improving drug regulation, and above all the appropriate use of this medication in athletic populations.

## Methods

The present study was a prospective, monocentric, randomized, sex-stratified, double-blinded, placebo-controlled, balanced, four-way cross-over phase I clinical trial (registered at Eudra CT with the number: 2015-005598-19 and DRKS with number DRKS00010574). The study was approved by the ethics committee of Ulm University (number 64/19) and was performed in accordance with the Declaration of Helsinki [33]. All participants gave written informed consent to participate in this study.

### Study Population

Inclusion criteria were (1) age 18–45 yrs., (2) endurance trained (V̇O_2max_ ≥ 52 ml/kg/min for males, ≥ 42 ml/kg/min for females; V̇O_2max_ measured on a bicycle ergometer) and (3) a personally signed and dated consent form before any study-related treatments or examinations could take place. Exclusion criteria included (1) females with a positive pregnancy test on enrolment or prior to investigational product administration, (2) allergy/hypersensitivity, (3) contraindication or (serious) adverse reaction of any component of the trial medication, (4) adverse medical history and concurrent disease, (5) participants who were incapable of giving informed consent and (6) a positive methacholine challenge test. Details on the exclusion criteria can be found in the study protocol published by Zügel et al.[34].

Pre-screening was conducted by asking potential participants about their endurance sport experience and recent competition race results. Selecting from a list of the highest-ranking participants, a total of 33 male and female participants were screened. Out of them, 25 individuals met the necessary criteria and were enrolled for the treatment phase. Due to protocol violations, one subject dropped out after the first study arm and was replaced.

### Medication

**Fig. 1:**
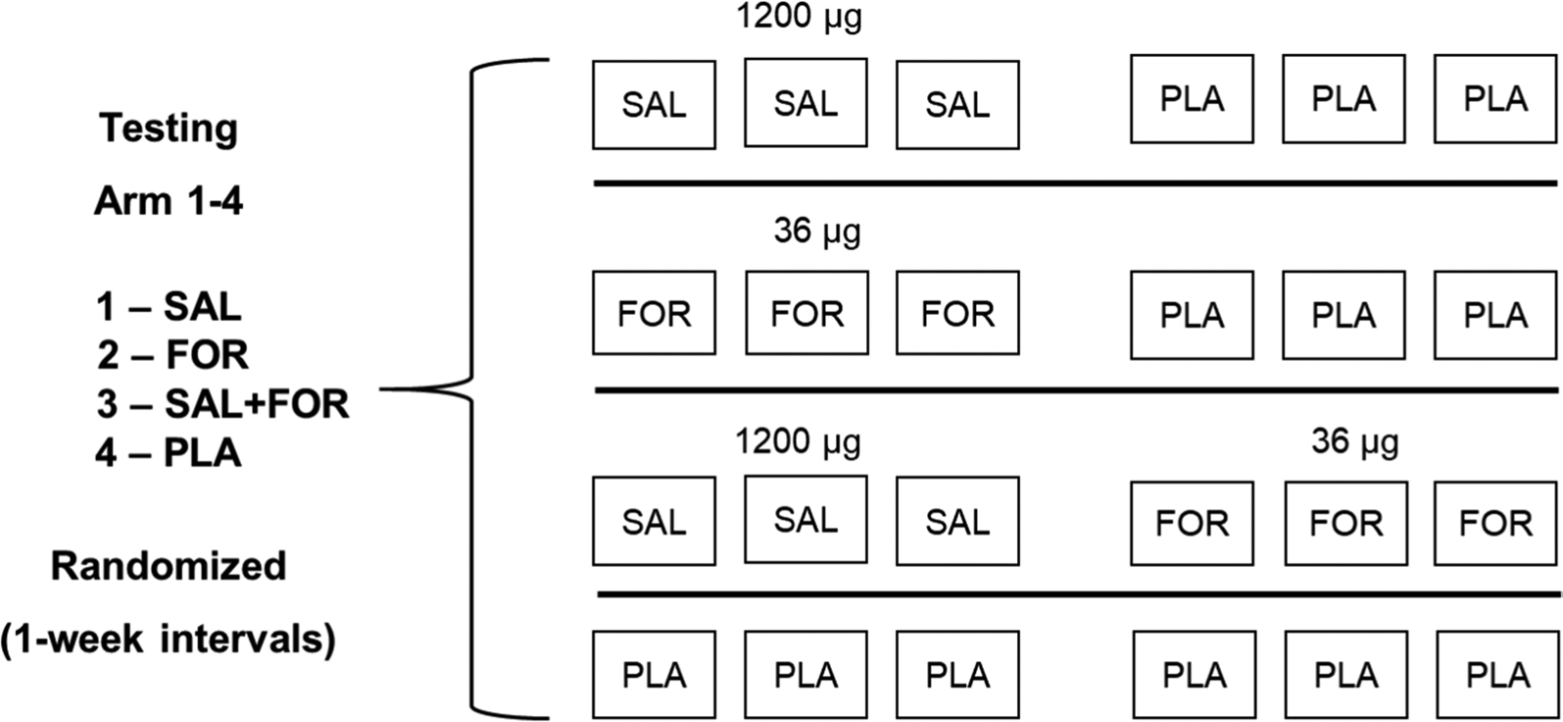
Schematic representation illustrating the randomized study medication scheme in the four study arms, separated by one-week intervals. Each participant received four blinded medication kits containing 24 sprays in total. Salbutamol (SAL), Formoterol (FOR), and Placebo (PLA)

The study medication was prepared and packaged in the central pharmacy at University Hospital Ulm. Each medication test kit consisted of six blinded powder inhalers, which were medication (1200 µg Salbutamol SAL [Cyclocaps, Pb Pharma GmbH, Meerbusch, Germany], 36 µg Formoterol FOR [Sandoz, HEXAL AG, Holzkirchen, Germany]) and/or placebo (PLA [Gelatine-coated capsules for dry powder inhaler containing lactose monohydrate, Pharmacy of the University Hospital Ulm, Germany]) (Fig. 1). These were provided in blinded packages for the four study arms for each participant (four medication kits per participant containing 24 sprays in total).

### Study Design

The detailed study design can be found in the published study protocol by Zügel et al. [34].

In short, the study design consisted of an (a) screening and (b) testing phase. Both, the screening phase and each study arm in the testing phase comprise two days for exercises and measurements.

The screening phase included preliminary performance and physiological testing to determine study participation eligibility. The testing consisted of a medical examination where the individual medical history was recorded, and anthropometric measurements, measurements of blood pressure (BP), heart rate (HR), echocardiography and 12-lead electrocardiogram (ECG) at rest, blood and urine collection, respiratory testing including several standardized variables like vital capacity, forced expiratory volume in 1 s, total lung capacity, reserve volume, specific airway resistance, etc., measured by a whole-body plethysmograph (COSMED, Rome, Italy), a methacholine (Provokit^®^ 0.33%, Aristo Pharma GmbH, Berlin, Germany) bronchial challenge test and a cardiopulmonary exercise test (CPX; including a ramp and verification test for V̇O_2max_ determination) were completed. A time trial (TT) for familiarization purposes of the participants on a bicycle ergometer followed by cardiac output measurements (*Q̇*) was also performed.

**Fig. 2:**
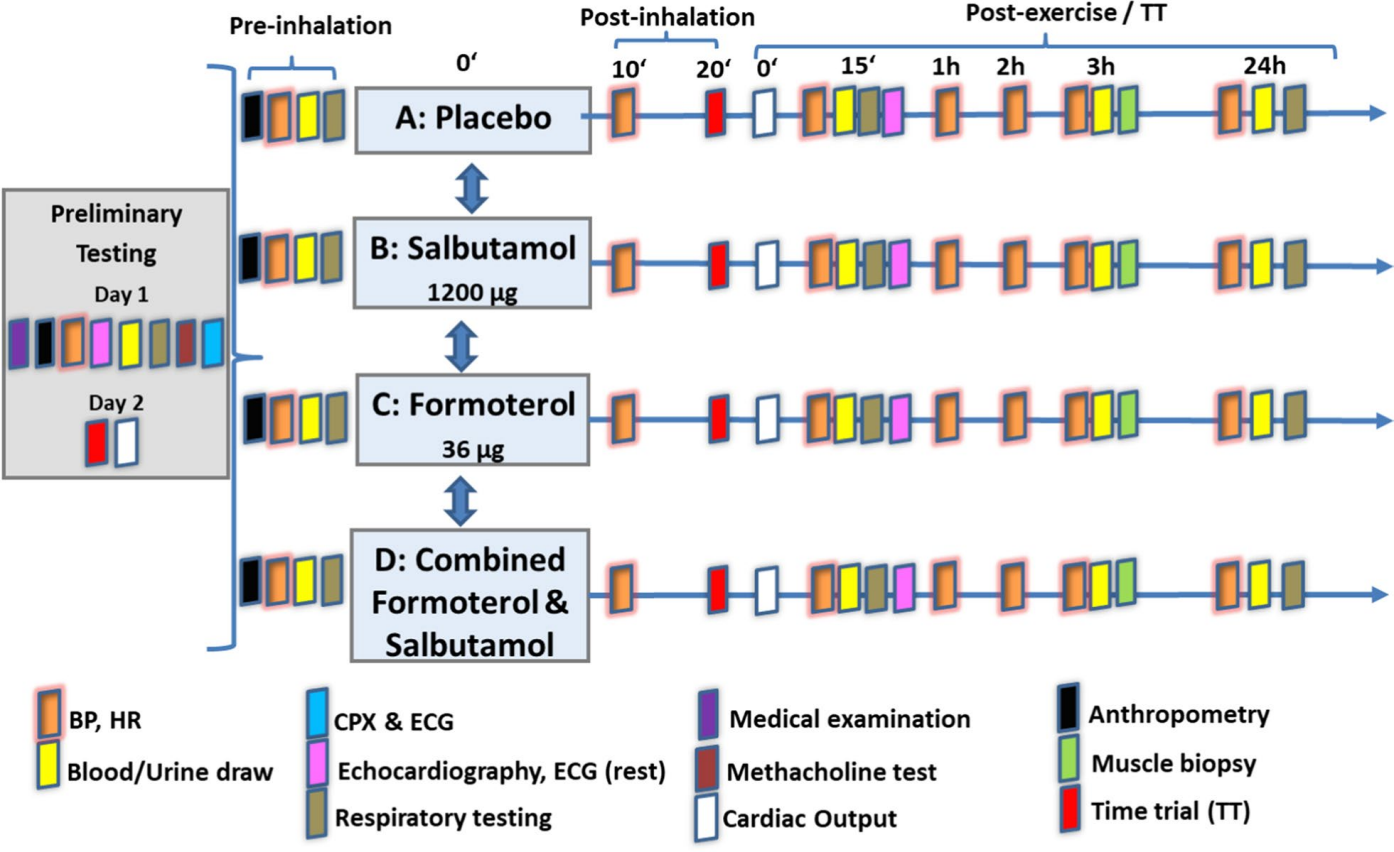
Study participants completed four study arms after the initial preliminary testing (screening for inclusion criteria + time trial (TT) familiarization). On the testing days—20 min after the participants inhaled the study medication—the TT procedure started (15 min warm-up followed by a 5-min low-intensity interval and the 10-min TT), where participants cycled 10 min at the highest possible workload. Blood and urine were collected before, directly after as well as 3 h and 24 h post-TT, and respiratory testing was performed at 3 h and 24 h post-TT. A muscle biopsy was collected 3 h after the TT. The time between study arms was 5–8 days. BP: blood pressure; HR: heart rate; ECG 12-lead electrocardiogram (ECG); CPX cardiopulmonary exercise test. Adapted from Zügel et al. [34]

The intervention phase with each of the four study arms (SAL/FOR/SAL + FOR/PLA) started with anthropometric measurements, BP and HR recordings at rest, followed by blood and urine collections and respiratory testing (Pre-TT). Afterwards, participants inhaled the study medication (powder inhalers), BP and HR were measured, and respiratory testing was conducted 10 min after application of the medication. The TT procedure started 20 min after the inhalation of the respective medication, beginning with a 15-min warm-up at 50% of the respective individual PV̇O_2_max. After the subsequent 5-min low-intensity interval at 100 W, the 10-min TT started. Here, participants targeted for the individually highest possible average mechanical power output per 10 min. Initial mechanical power output was equal to 90–95% of maximum mechanical power output obtained in the preliminary CPX. During the TT, *Q̇* and HR were measured continuously and non-invasively (Clearsight^®^, Edwards LifeSciences, Irvine, CA, USA) via pulse contour and volume clamp. The variables BP, HR, respiratory testing, and echocardiography were recorded 15 min after the end of the TT (Post-TT). Furthermore, HR and BP were also measured 1 h, 2 h, 3 h (3 h Post-TT) and 24 h (24 h Post-TT) after the end of the TT. Blood and urine were collected, and respiratory testing was performed at 3 h and 24 h post-TT. A muscle biopsy was collected 3 h after the TT. Additionally, at day 3 or 4 the participant was asked for adverse events via telephone. The time between study arms was 5–8 days. An overview of the procedures and time points of their assessment is provided in Fig. 2 and Additional file 1: Table S1.

### ***Urine and Serum Levels of ***β***2-Agonists Measured by UHPLC-MS/MS***

Urinary and serum concentrations of salbutamol and formoterol were determined by ultra-high-performance liquid chromatography hyphenated to tandem mass spectrometry (UHPLC-MS/MS). As concentrations are expected to be at ultra-trace levels from literature reports [35], a triple quadrupole mass analyser was utilized. Urine analysis was possible using dilute-and-inject, while serum analysis required solid-phase-extraction for sample preparation.

### Muscle Biopsy

In previous experiments, we determined that maximum expression of target genes like exercise-induced NR4A were found at highest expression rates after 3 h post-exercise [36]. Thus, the skeletal muscle biopsy took place 3 h post-TT using the Bergström technique according to published protocols [37]. When the participant was suitable for biopsy, skeletal muscle samples were obtained under local anaesthesia from the *Musculus vastus lateralis* (femur) of the dominant side of the participants approximately 20 cm above the knee. Participants could walk and train at the same day and were fully able to use their legs.

To isolate RNA, muscle tissue was incubated for 24 h with RNAlater (QIAGEN GmbH, Hilden, Germany) at 4 °C and then stored in cryotubes at − 80 °C until further analysis. Muscle tissue for protein examination was immediately cryopreserved with liquid nitrogen and stored at − 80 °C until further analysis.

### Microarray Analysis

To determine relative expression on the mRNA level of the different medication arms compared to Placebo, a pathway and expression analysis with the human array chip Clariom S (Thermo Fisher Scientific, MA, USA) and the Transcriptome Analysis Console (TAC) 4.0 Software (Thermo Fisher Scientific, MA, USA) was performed. The microarray platform used was Affymetrix^®^ Clarion S gene array chip. After hybridization, gene transcription was analysed. Following normalization, differential expression was carried out using eBayes function and one-way repeated measures ANOVA statistical analysis. Gene-level fold changes were analysed at <  − 1.5 and > 1.5 and *p *≤ 0.05. The NR4A family was specifically analysed independent of fold change or significance level. Pathways provided by WikiPathways were used for further analysis. Genes above the threshold were sorted by count and significance and up-regulation/down-regulation visualized in the pathways. The provided pathways were manually screened for pathways involved in response to exercise. Specific target genes with high fold changes were further examined.

### RT-PCR

Muscle biopsy samples of the participants were examined regarding gene expression of NR4A1/NR4A2/NR4A3. RNA was quantified with spectrophotometry (NanoDrop 2000c, Thermo Scientific, Massachusetts, USA) and transcribed to cDNA with the QantiTect^®^ Reverse Transcription Kit (QIAGEN GmbH, Hilden, Germany) according to the manufacturer's instructions. The cDNA was used to determine the expression with real-time qPCR (RT-qPCR) analogous to established protocols [38] with GAPDH as established reference gene for endurance exercise [38, 39].

### Protein Preparation

To determine the respective NR4A protein abundance, muscle samples were incubated with 250 µl Pierce™ RIPA buffer (Thermo Fisher Scientific, MA, USA), mixed with cOmplete™ Mini EDTA-free Protease Inhibitor Cocktail (Roche, Basel, Switzerland), homogenized and incubated for 10 min on ice in Pierce™ RIPA buffer. After centrifugation (4 °C, 20,817 × g, 20 min), the supernatant was used for SDS-PAGE with a final concentration of 10 µg/µl sample according to the manufacturer's instructions (Mini-Protean TGX Gels 4–20%, BIO-RAD, Berkeley, USA).

### Hormonal Targets

Insulin-like growth factor-1 (IGF-1 ELISA (MD5801) Tecan Group Ltd; Männedorf, Switzerland), adrenaline (Human Adrenaline ELISA (MBS494515); MyBiosource; San Diego, CA, USA), noradrenaline (Human Noradrenaline ELISA (MBS161498); MyBiosource; San Diego, CA, USA) and transforming growth factor-β (TGF-beta 1 Human ELISA (BMS249-4); Invitrogen/Thermo Fisher Scientific; Waltham, MA, USA) concentrations were determined by enzyme-linked immunosorbent assay (ELISA) according to the manufacturers’ instructions.

Adrenocorticotropic hormone (ACTH), follicle-stimulating hormone (FSH), luteinizing hormone (LH), cortisol, insulin as well as n-terminal prohormone of brain natriuretic peptide (NT-BNP) concentrations were measured with the ElectroChemiLuminescence ImmunoAssay [ECLIA; Roche Cobas pro (e801 Modul); Basel; Switzerland] in accordance with clinical standards.

### Statistics

The detailed statistical analysis procedure can be found in the study protocol [34]. Besides the analysis of a treatment effect by the medication, further significant effects were examined regarding sex (possible effects caused by sex differences) and period (difference within the study arms 1–4, which may indicate, e.g. a training effect). The detailed data of the biometric analysis results including sex and period effects are mainly provided in the data repository OPARU of the Ulm University [40].

Statistical analysis was carried out using SAS, version 9.4, under Windows. All statistical tests were two-sided at a significance level of 5%. Because of the explorative nature of this study, no adjustment for multiple testing was done. All results from the statistical tests were regarded as hypothesis generating only, and not as proof of efficacy.

To determine the concentration differences of serum β2-agonist between males and females, a two-way ANOVA with following Tukey's multiple comparisons test was also additionally conducted with GraphPad Prism 9.5. (San Diego, CA, USA). GraphPad Prism 9.5. was also used for graphical representation of the data.

## Results

### β2-Agonist Urine and Serum Concentrations

Administration of the different β2-agonists and the respective combination or Placebo was reliably detected in urine even without previous unblinding (Additional file 1: Fig. S1).

**Fig. 3:**
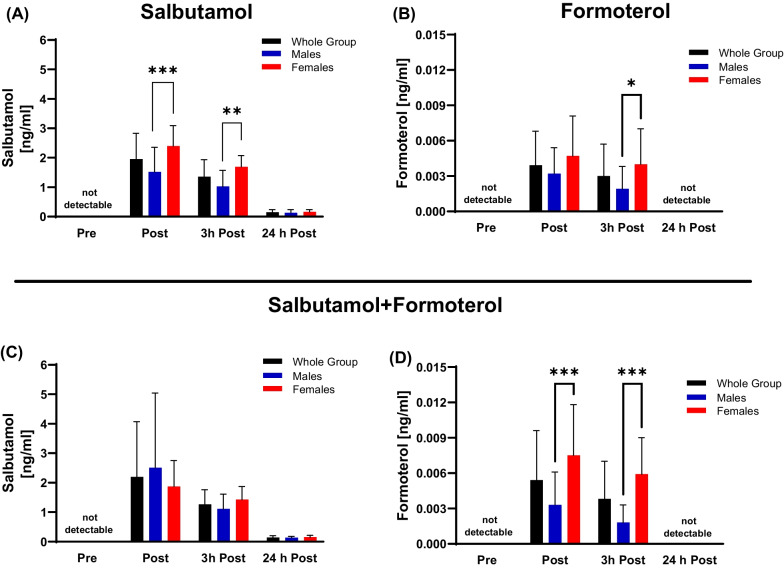
Concentrations of β2-agonists determined in serum for salbutamol, formoterol and the combined dosage salbutamol + formoterol in the whole group as well as separated in male and female participants. **A** Acute serum salbutamol inhalation resulted in a concentration increase that gradually decreased at 3 h post-TT and 24 h post-TT. The absolute concentration was higher in females compared to males. **B** Administration of formoterol showed the highest concentration post-TT in all participants and was decreased at 3 h with higher values observed in females. **C** The combined medication resulted in higher salbutamol concentrations for males compared to female participants post-TT, whereas at 3 h and 24 h post-TT females still had higher detectable salbutamol serum concentrations. **D** Formoterol concentrations in females were significantly higher after salbutamol + formoterol inhalation at post-TT and 3 h post-TT compared to their male counterparts. All data are presented as mean ± standard deviation. Significance set at *p ≤ 0.05; **p ≤ 0.01; ***p ≤ 0.001

Serum samples revealed a higher concentration of SAL (*p *< 0.001) for females compared to males post-TT. Conversely, all three medications (SAL *p *= 0.003, FOR *p *= 0.024, and combination SAL + FOR: FOR *p *< 0.001) resulted in higher β2-agonist concentrations in females 3 h post-TT, possibly to faster distribution in blood and longer systemic circulation in females besides absolute higher concentrations for SAL and FOR (Fig. 3A–D).

While SAL treatment was detected even after 24 h post-TT, FOR was only detectable in the post-TT and 3 h post-TT samples but not in serum after 24 h post-TT independent of sex. The latter observation was most likely due to the low dose of 36 µg that results in very low total blood concentrations (Fig. 3A–D).

### Anthropometry, Aerobic Capacity, Time Trial Performance and Lung Function Testing

In total, 24 participants (12 female/12 male) completed all four treatment arms. Anthropometric data and CPET testing of the participants indicated a high aerobic capacity reflected by mean V̇O_2max_ of 57.1 ± 6.2 ml/min/kg for female and 63.0 ± 5.0 ml/min/kg for male participants (Table 1).

**Table 1:** Anthropometric and performance data of the ELSA participants (*N *= 24, 12 female/12 male)

	Female	Male
Age (years)	22.92 ± 2.72	24.42 ± 4.55
Standing height (cm)	170.82 ± 5.55	180.45 ± 3.74
Body mass (kg)	62.16 ± 5.03	73.94 ± 5.54
V̇O_2max_ (ml/min/kg)	57.1 ± 6.2	63.0 ± 5.0
PV̇O_2_max (W)	305 ± 30	417 ± 25

All data are presented as mean ± SD

*V̇O*_*2max*_ Maximum oxygen uptake, *PV̇O*_*2max*_ Power at V̇O_2max_

### TT Performance

**Fig. 4:**
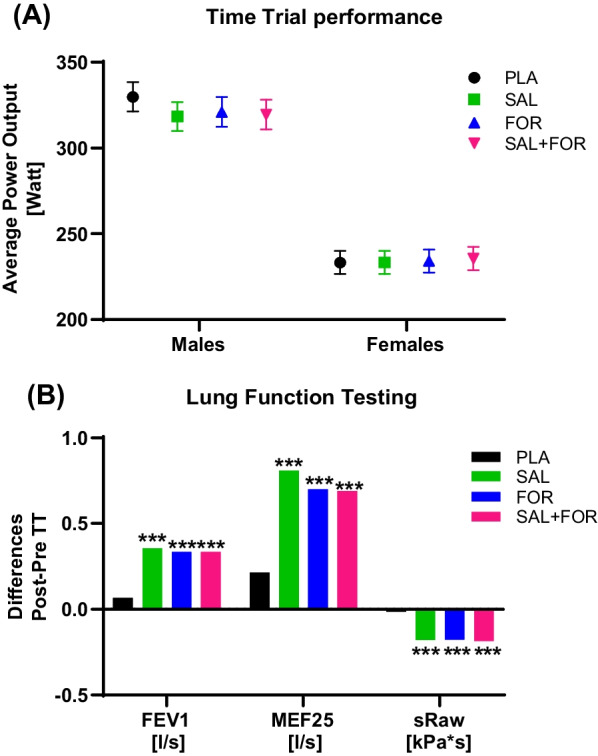
**A** Time trial performance represented by average power output and **B** selected lung function variables (differences) by treatment with salbutamol (SAL), formoterol (FOR), salbutamol + formoterol (SAL + FOR) as well as placebo (PLA) during the 10-min-long time trial test. No performance-enhancing effect was observed for all medications in the average power output for males or females. Lung function variables—represented by FEV1 (volume that has been exhaled at the end of the first second of forced expiration), MEF25 (Mean expiratory flow) and sRaw (specific resistance of the airways)—significantly improved for all β2-agonist combinations compared to PLA. Data are presented as mean ± standard error for average power output. ***p ≤ 0.001 versus PLA

No treatment effect of the acute medication of SAL, FOR or Sal + FOR compared with PLA on TT performance was detected. The average mechanical power output of the male participants did not significantly differ between the study arms neither in male nor in female participants (Fig. 4A). Sex and periodic effects for the whole group (all participants analysed together) and male participants were observed for peak power without evidence for a treatment effect or performance enhancement (Table 2).

**Table 2:** Time trial as well as lung function variables and statistical outcomes

Variable	Treatment effect	Sex effect	Period effect
*Time Trial performance*			
TT APO [W]	–	wg	–
TT RPO [%]	–	wg	–
Peak Power [W]	–	wg	wg/m
*Lung function testing*			
FVC % predicted	–	–	f (24 h Post–Pre-TT)
FEV1 [l/s]	Wg/m/f (Post–Pre-TT)	–	–
FEV1% predicted	wg/m/f (Post–Pre-TT)	–	–
MEF 25 [l/s]	wg/m/f (Post–Pre-TT)	–	–
MEF 25 predicted	wg/m/f (Post–Pre-TT)	–	f (Post–Pre-TT)
TLC [l]	–	–	f (Post–Pre-TT)
TLC % predicted	–	–	f (Post–Pre-TT)
sRaw [kPa*s]	wg/f (Post–Pre-TT)	–	–

No treatment effect was found for any variable of time trial performance, while sex affected each of these variables

*TT* Time trial, *APO* Average power output, *RPO* Relative power output, *FVC* Forced vital capacity, *FEV1* Volume that has been exhaled at the end of the first second of forced expiration, *TLC* Total lung capacity, *MEF* Mean expiratory flow, *sRaw* Specific resistance of the airways, *24 h-/Post–Pre-TT* Data compared between the lung function test time before and after the TT, *Wg* Whole group, *f* Female, *m* Male

### Lung Function Testing

A treatment effect of the different medications was observed for males and females as well as the whole group post-TT compared to the lung function test pre-TT. Increasing lung function (FEV1, MEF25 and sRaw) was detected (Fig. 4B) without clinically relevant side effects (e.g. trembling, nervous tension, headaches, palpitations, muscle cramps etc., asked after the respective medication inhalation and at a telephone call three days after each TT).

Except for total lung capacity, a treatment effect was measured for the whole group as well as male and female participants for the examined lung function variables. While no sex effect was observed, analysis revealed period effects for females Post–Pre-TT and 24 h Post–Pre-TT. An overview of the effects for variables assessed in the lung function test is provided in Table 2.

### Effect of Time Trial and Medication on Echocardiographic Variables

Left ventricular end-systolic volume (ESV) showed a significant treatment effect (*p *= 0.002) in females, but there was no significant treatment effect (*p *= 0.660) in males. Regarding the whole group, this results in a significant treatment effect (*p *= 0.037) as well as a sex effect (*p *= 0.002) (Table 3).

**Table 3:** Summary of the treatment, sex and period effects of the post-interventional strain analysis for the left ventricle Post–Pre-TT. Statistical significance was set at *p *≤ 0.05

Variable	Treatment effect	Sex effect	Period effect
EDV (ml)	–	wg	–
ESV (ml)	wg/f	wg	m
EF (%)	wg/f	–	m
EndoGLS	wg/f	wg	–
MyoGLS	wg/f	wg	–
GRS	wg	–	–

Statistical significance was set at *p *≤ 0.05

*EDV* End-diastolic volume, *ESV* End-systolic volume, *EF* Ejection fraction, *EndoGLS* Endocardial global longitudinal strain, *MyoGLS* Myocardial global longitudinal strain, *GRS* Global radial strain, *Wg* Whole group, *f* Female, *m* Male

Endocardial Global Longitudinal strain (EndoGLS) is a variable for the global positional change and thus the contractility of the left ventricular endocardium. It is thus a measure of the contractility of the left ventricle. A treatment effect for the whole group as well as for the female participants was observed for EndoGLS and myocardial GLS. In males, significance was only evident for SAL + FOR versus PLA (*p *= 0.047), whereas in females, SAL (*p *= 0.010), FOR (*p *< 0.001) as well as SAL + FOR (*p *< 0.001) were each significant.

### Microarray analysis/Gene and Protein Expression of NR4A Family

**Fig. 5:**
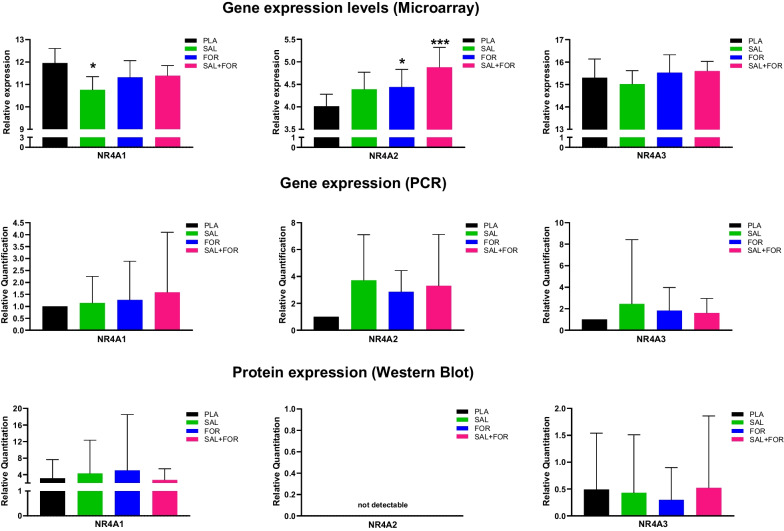
NR4A-family (NR4A1/NR4A2/NR4A3) gene and protein expression for the different study arms placebo (PLA), salbutamol (SAL), formoterol (FOR) as well as salbutamol + formoterol (SAL + FOR). (Top) Microarray analysis revealed increased relative expression of NR4A2 for FOR and SAL + FOR compared to PLA, while SAL treatment resulted in decreased NR4A1 expression. (Middle) Although the gene expression of the medication SAL, FOR and SAL + FOR was higher than PLA (normalized to 1) for all NR4As, no statistically significant difference was determined. (Bottom) Protein expression did not differ between the study arms for NR4A1 and NR4A3. NR4A2 protein was not detected in the muscle samples. *All data are presented as mean standard deviation. Significance level set at *p ≤ 0.05 and ***p ≤ 0.001 versus PLA

Microarray analysis revealed a significant treatment effect for NR4A2 (relative gene expression: PLA 4.01, SAL 4.39, FOR 4.44, SAL + FOR 4.88; *p *= 0.007) with the highest effect between PLA and SAL + FOR (*p *= 0.001), while no significant treatment effect on gene expression of NR4A1 or NR4A3 was observable (Fig. 5).

Similarly, no differences in expression between the study arms for NR4A1 and NR4A3 was observed following PCR analysis of NR4A family gene expression. A significant treatment effect was observed for NR4A2, but only in 13 of 24 participants NR4A2 gene products were detected (Fig. 5).

Protein expression of NR4A family in muscle revealed a sex effect in NR4A3 (*p *= 0.0357) for the whole group. No other effects (treatment, sex and period) effects were observed for NR4A1 or NR4A3, while no NR4A2 protein was detected in all groups (Fig. 5).

### Additional Microarray Analysis

The combination of β2-agonists influenced up- and down-regulation of differently expressed genes most compared to the other study arms (23 genes in total). Further pathway analysis with TACx and linked WikiPathways revealed treatment effects in the following energy metabolism related genes: ATF3 (e.g. hypertrophy model; TGF-beta signalling pathway), PDK4 (e.g. oestrogen receptor pathway; nuclear receptors meta-pathway), LPL (e.g. metabolic pathway of LDL, HDL and TG; PPAR signalling pathway), CREM (e.g. mBDNF and proBDNF regulation of GABA neurotransmission) and ATP1B3/ATPase (e.g. calcium regulation in cardiac cells) (Additional file 1: Table S2).

### Hormonal Targets

The hormones IGF-1, TGF-β, noradrenaline, adrenaline LH, FSH, ACTH, cortisol, insulin, glucagon and proNT-BNP were analysed in serum. Here, IGF-1 (period effect wg), FSH (decreased SAL + FOR 3 h post-TT compared to PLA), LH (3 h post-TT) and insulin concentrations (decreased post compared to pre) showed treatment effects, while for TGF-β, adrenaline, noradrenaline, ACTH, cortisol, proNT-BNP and glucagon, no treatment effect was observed. All other effects are described in Table 4. A graphical representation of the hormonal data is provided in Additional file 1: Fig. S2.

**Table 4:** Summary of the treatment, sex and period effects of the hormonal target analysis Post–Pre-TT, 3 h Post–Pre-TT and 3 h Post–Post-TT

Variable	Treatment effect	Sex effect	Period effect
Adrenaline [ng/ml]	–	–	wg/f (Post–Pre-TT)
Noradrenaline [ng/ml]	–	–	–
TGF-β [ng/ml]	–	–	–
IGF-1 [ng/ml]	–	–	wg
ACTH [ng/ml]	–	wg	–
NT-BNP [pg/ml]	–	wg	–
Cortisol [µg/dl]	–	–	–
Insulin [mU/l]	wg (Post–Pre-TT)	–	–
FSH [IU/l]	wg/f (3 h Post–Post-TT)	–	
LH [IU/l]	wg/f (3 h Post–Pre-TT)	–	–

*TGF-*β Transforming growth factor-β, *IGF-1* Insulin-like growth factor-1, *ACTH* Adrenocorticotropic hormone, *NT-BNP* N-terminal prohormone of brain natriuretic peptide, *FSH* Follicle-stimulating hormone, *LH* Luteinizing hormone, *Wg* whole group, f Female, *m* Male, *3 h-/Post–Pre-TT* data compared between the lung function test time before and after the TT

## Discussion

The present study examined the 10-min TT performance after acute high doses of β2-agonists SAL, FOR and their combined application SAL + FOR in healthy female and male endurance athletes, including potential effects on the respiratory, cardiac, hormonal, gene and protein expression level.

Higher serum β2-agonists concentrations were observed in females as well as improved lung function and hormonal changes in FSH, LH, insulin and IGF-1 (only females) without any significant influence on TT performance. A clear up-regulation of the performance-enhancing NR4A family on the protein level was not detected, while genetic analyses by microarray revealed the highest induction with SAL + FOR of NR4A and concomitantly high interindividual responses.

### TT Performance

Performance was not enhanced by the applied acute and single dose β2-agonist combinations compared to PLA during a 10-min TT at high to severe intensity, although an acute and significant effect of improved flow-related measures of lung function, in particular FEV 1, MEF 25 and sRaw, was observed in our non-asthmatic participants.

A recent meta-analysis conducted by Riiser et al. did not reveal any effect of β2-agonists on aerobic performance in non-asthmatic participants regardless of type, dose, administration route, duration of treatment or performance level of participants [15]. Conversely, another meta-analysis by the same group, including 34 studies examining the effects of β2-agonists on strength, sprint and anaerobic performance showed that this dimension of performance enhancement was higher with repeated dosing over several weeks than with single dosing [14]. In general, the effect of supratherapeutical doses was judged to be clearly performance-enhancing, but the group was unable to make a clear statement on the extent of the effect within the permitted range against the background of their analyses, since the results of the examined studies varied [14]. On the cellular level, the investigated β2-agonists SAL and FOR mediate various potentially performance-enhancing effects. They increase lean muscle mass, protein metabolism and protein biosynthesis after strength training and the oxidation of fatty acids as well as lipolysis in various human studies [41], while the exact dosage and frequency of application that are necessary to exert these effects to relevant proportions could yet not conclusively be established. Thus, within the permitted range, performance enhancement seems unlikely. However, in competitive sports even small increases in performance gain can be decisive between win and loss.

### Lung Function and Muscular Hypertrophy

Lung function was significantly affected by the treatment, and all medications improved FEV1 (amount of air forceable from the lungs in one second), MEF25 (maximal expiratory flows at 25% of FVC) and sRaw (the work related to changes in lung volume while overcoming a fixed resistance) in our healthy, non-asthmatic participants. Conversely, TT performance did not improve due to this higher lung function. Interestingly, although endurance training induces large and significant adaptations within the cardiovascular, musculoskeletal and haematological systems, the structural and functional properties of the respiratory system do not adapt in the same way in response to repetitive physical exercise [42]. Several studies examined the respiratory adaptions of athletes either in aerobic or anaerobic sports, but found no differences in pulmonary characteristics (spirometric function values, diffusion capacity) in athletes compared to non-athletes at rest or between different types of sports [43, 44]. Furthermore, even intranasal stent applications for improved breathing during intense exercise did not show any improvement in exercise performance [45], so the contribution of the observed dilatory effect in non-asthmatic lungs on performance capacity seems neglectable. As the asthmatic prevalence in elite athletes is higher than in the general population [7, 9, 42] and consequently the chronic use of β2-agonists common, the possible short- and long-term application in the affected athletes may be different to their healthy peers.

But while there seems no performance-enhancing effect by improved lung function, β2-agonists can exert anabolic and lipolytic functions on the molecular level, the extend depending on dose and route of administration [16]. In contrast to our acute one-dose administration, Hostrup et al. [41] examined the chronic use of daily therapeutic inhalation of β2-agonist in healthy young males and found increases in insulin-stimulated whole-body glucose disposal during a four-week application period, which was associated with an increase in lean mass. We did not especially examine hypertrophic signalling in our muscle samples, but found up-regulated genes like the ATF3 involved in hypertrophic stimulation. Although the underlying mechanism of the hypertrophic signalling could not be fully elucidated in the study by Hostrup or with our microarray data, the NR4A family may be proposed as one contributor for the observed metabolic and muscular adaptations [19, 46].

### NR4A Family and Hormonal Targets

The NR4A expression on the genetic and protein level was not altered with additional β2-agonist use compared to PLA. The NR4A family belongs to the orphan receptor family and includes three members, namely Nur77 (NR4A1), Nurr1 (NR4A2) and Nor1 (NR4A3) [47]. Their expression is involved in regulation of the expression of genes which participate in several biological functions, including metabolism (particularly glucose and fatty acid utilization genes in skeletal muscle), immunity, cellular stress, memory and insulin sensitivity [36, 47, 48]. As early-response genes without endogenous ligands, their expression is induced by diverse stimuli, e.g. exercise-related activators of cAMP and protein kinase signalling, mechanical stress or physiological activity [49, 50]. We and others showed in previous studies an increase in the NR4A receptor family expression after exercise, peaking at around 3 h [36, 51].

There is an increase in scientific evidence suggesting that significant changes in metabolic and trophic programs at the core of skeletal muscle plasticity via the NR4A subgroup are adaptively regulated by adrenergic stimulation, involving adrenergic receptor activation, turnover, and downstream signalling [19, 20, 46]. Up-regulated NR4A expression was observed after acute one-legged [52] and sprint [51] exercise. The present study did not confirm a direct influence of β-adrenergic stimulation and resulting increased expression, which may be due to the superimposing effect of the TT that itself induces NR4A expression, and the β2-agonists effect may be blunted. A basal resting muscle biopsy before the TT might have resulted in different conclusions, but that can only be speculated. In addition, although the microarray analysis in a participant subgroup showed significant increases for NR4A2 compared to PLA, PCR confirmed these results only in a limited number of participants. Gene expression of NR4A2 was observed to be very low with a mean Ct-value > 35 and conclusions about significant up-regulation should be interpreted with caution as these results were not confirmed on the protein level. Here, no increase in NR4A by β2-agonists compared to PLA was observed. Although NR4As are early-response genes, the time frame between medication application and the muscle biopsy three hours later may be too short to detect significant increases in the protein concentration.

### Hormonal Targets

To examine the activation of the β2-adrenergic receptor pathway and potential performance-enhancing molecular interactions, several hormonal markers were assessed. The analysed circulating hormonal targets ACTH, NT-BNP, cortisol, IGF-1, TGF-β, noradrenaline and adrenaline showed no β2-agonists related effects, but high interindividual variation of concentrations in our cohort may contribute to this result as well as the presumably only minor impact of the β2-agonists on these parameters.

Adrenaline and noradrenaline are the main catecholamines for β2-receptor binding and activation [53]. Although acute high-intensity exercise normally increase adrenaline and noradrenaline release [54], we did not observe any significant changes of both catecholamines in plasma after the TT in all study arms. Thus, an additional stimulation besides the medication by increased catecholamine production seems unlikely. The included test TT during the screening phase may have contributed to these results, as the participants—well trained and competitive athletes—were accustomed to the TT procedure potentially resulting in subsequently lower stress response underlined by unchanged cortisol levels at all time points.

In contrast, treatment effects were observed for insulin, FSH and LH. As mentioned above, increased insulin sensitivity and glucose disposal were observed after chronic β2-agonist use [41], which would explain our results of decreased insulin values after the TT. Although the acute exercise itself contributes to increased insulin uptake and sensitivity [55], the statistical evaluation showed a clear treatment effect for the medications compared to PLA in our study, underlining the influence of the β2-agonists on energy metabolism with the used doses. Albeit there is no direct connection between the β2-adrenergic pathway and sexual hormone secretion, the anabolic effect of increased LH and subsequently downstream testosterone release may exert potential performance-enhancing effects [56] by molecular mechanisms currently unknown. Interestingly, both FSH and LH exhibited a treatment effect measurable for the whole group and not only sex-specific, which shows that other influences besides different hormonal regulation may be relevant. The measurement of the circulating testosterone/LH ratio in our participants may reveal further clues of the interaction between the glucocorticoid and β-adrenergic pathways.

To minimize any further influence by the different sex-specific hormonal responses besides our statistical correction approach, we assessed the time of menstrual cycle in our female participants. We did not find any interdependence between time of menstrual cycle phase and physical performance or endocrine parameters, which strengthens the observed results, especially as no increase in cortisol levels were measured.

### ***Effect of ***β***2-Agonists on Cardiac Contractility***

We examined cardiac changes after medication and TT by echocardiography. The detailed analysis of all variables with focus on the left ventricular global longitudinal strain (describes the deformation of the cardiac wall), which is considered one of the most robust and valid clinical parameter [57], is described in another work of our group (unpublished results). In short, EF was increased by all medication variants, while ESV showed statistical significance in females induced by the medication. For endocardial strain, the results also differed between males and females. There was a treatment effect on all these parameters, as well as on the myocardial and radial strain. This suggests an increased contraction of the heart in all recorded dimensions (global longitudinal shortening and radial myocardial thickening).

Regarding the analysis of individual drug administrations, the effect was stronger with formoterol. In addition, the highest effect was observed for the females' cohort. This may be due to the fact that females have a significantly lower body weight and a higher body fat percentage. This results in a lower fat-free mass, i.e. a significantly reduced distribution volume for the hydrophilic active substances. Due to the confirmed higher serum levels reached on average in our tested female athletes, females may possibly benefit from sex-unspecific maximum doses in sports, at least with regard to β2-agonists use. This observation raises the questions if (a) the drug dosage may be adjusted to the subject-specific fat-free mass or body surface area, (b) the resulting serum levels should be routinely measured in anti-doping analyses and (c) if consequently individual sex-specific threshold may be implemented.

In conclusion, the result that in particular the inotropy of the heart is significantly affected by the β2-agonists inhaled administration underlines that these pharmaceutical agents are also potent drugs with systemic effects and side effects with clear differences between the sexes at the same dosage.

### Limitations

The acute doses of SAL (1200 µg) and FOR (36 µg) were lower than the doses allowed within 24 h [SAL 1600 µg (divided into 600 µg/8 h as of 2023)/FOR 54 µg] according to the WADA list of prohibited substances. A basal muscle biopsy before administration of the different medications or the TT would have been a valuable asset to distinguish between effects caused by the exercise test versus medication.

The analysis of haemodynamic variables, especially *Q̇* and stroke volume, would have provided interesting insights into the mechanisms of a potential change in performance, which is why we had included a non-invasive pulse contour measurement into the study protocol, promising a valid assessment without perturbation of the TT [58]. Unfortunately, the Clearsight^®^ system used proved to be unreliable in many subjects in the present study, and we thus were not able to include these data here. However, this issue seems less critical due to the lack of performance effects.

A further limitation of our study is that the participants were not elite athletes. However, due to WADA regulations, it was not possible to include elite athletes, and we recruited 24 female and male athletes of the highest available level, all of them highly endurance trained and experienced competitors.

Nevertheless, the study design of our double-blinded randomized control trial allowed us to draw comparative causal treatment inferences, minimizes allocation, selection, and assessment bias, as well as minimize confounding factors by randomization.

## Conclusion

There is most likely no performance-enhancing effect on 10-min TT performance in moderate to highly trained athletes with the used single doses of β2-agonists either alone (SAL or FOR) or in combination (SAL + FOR) compared to PLA. Microarray analysis of a subgroup of participants revealed significant up-regulation with combined doses of SAL + FOR of genes involved in energy metabolism, hypertrophy and signal transduction that might give further valuable perspectives and targets for the examination of the performance-enhancing effects of combined use of β2-agonists.

As the doses of the different medications were equal for all participants, irrespective of body mass or sex, it needs to be clarified whether sex-specific differences in, hormonal and cardiac effects can possibly be explained by the different serum levels and body composition or whether other mechanisms such as sex-specific differences in receptor expression and sensitivity might play a role.

### Supplementary Information

**Additional file 1: Table S1.** Schedule illustrating interventions of the ELSA trial. **Fig. S1.** β2-Agonist (salbutamol (SAL)/formoterol (FOR)/combination SAL+FOR) detection in urine samples for each study arm (A1-A4) by LC-MS. The different medication application could be reliable detected and assigned to the respective study arm. An example analysis is given for one participant, where A1 (PLA), A2 (SAL+FOR), A3 (SAL) and A4 (FOR) could be assigned without unblinding. **Fig. S2.** Hormonal response of the participants before (Pre) β2-agonist (salbutamol (SAL)/formoterol (FOR)/combination SAL+FOR) application, directly after the time trial (Post) and 3 h after the time trial (3h Post) in blood serum. The different medication application resulted in a treatment effect for the whole group for (F) follicle-stimulating hormone (FSH), (H) insulin and (J) luteinizing hormone. No treatment effect was observed for (A) adrenaline, (B) noradrenaline, (C) tumour-growth-factor beta (TGF-beta), (D) insulin growth factor 1 (IGF-1), (E) adrenocorticotropic hormone (ACTH), (G) n-terminal prohormone of brain natriuretic peptide (NT-BNP) or (I) cortisol. Statistical significance was set at #<0.05 for the treatment effects between the different time points. **Table S2.** Summary of 23 most up-regulated gene expression for SAL+FOR determined by microarray. Genes involved in exercise and metabolism are marked in bold.

## Data Availability

The data that support the findings of this study are openly available in OPARU at http://dx.doi.org/10.18725/OPARU-47213.

## Abbreviations

SAL: Salbutamol
FOR: Formoterol
PLA: Placebo
TT: Time trial
AHR: Asthma/airway hyper-responsiveness
TUE: Therapeutic use exemption
SABA: Short-acting β2-agonist
ICS: Inhaled corticosteroids
LABA: Long-acting β2-agonists
WADA: World Anti-Doping Agency
NR: Nuclear hormone receptor
*Q̇*: Cardiac output
V̇O_2max_: Maximum oxygen uptake
BP: Blood pressure
HR: Heart rate
ECG: 12-lead electrocardiogram
CPX: Cardiopulmonary exercise test
