# Supplementary material for: Acute Effects of Single Versus Combined Inhaled β2-Agonists Salbutamol and Formoterol on Time Trial Performance, Lung Function, Metabolic and Endocrine Variables
Source: Sports Med Open. 2023 Aug 28;9:79. doi: 10.1186/s40798-023-00630-3 (PMC10462601; doi:10.1186/s40798-023-00630-3)
Supplement: Supplementary file 1 — Additional file 1: Table S1. Schedule illustrating interventions of the ELSA trial. Fig. S1. β2-Agonist (salbutamol (SAL)/formoterol (FOR)/combination SAL+FOR) detection in urine samples for each study arm (A1-A4) by LC-MS. The different medication application could be reliable detected and assigned to the respective study arm. An example analysis is given for one participant, where A1 (PLA), A2 (SAL+FOR), A3 (SAL) and A4 (FOR) could be assigned without unblinding. Fig. S2. Hormonal response of the participants before (Pre) β2-agonist (salbutamol (SAL)/formoterol (FOR)/combination SAL+FOR) application, directly after the time trial (Post) and 3 h after the time trial (3h Post) in blood serum. The different medication application resulted in a treatment effect for the whole group for (F) follicle-stimulating hormone (FSH), (H) insulin and (J) luteinizing hormone. No treatment effect was observed for (A) adrenaline, (B) noradrenaline, (C) tumour-growth-factor beta (TGF-beta), (D) insulin growth factor 1 (IGF-1), (E) adrenocorticotropic hormone (ACTH), (G) n-terminal prohormone of brain natriuretic peptide (NT-BNP) or (I) cortisol. Statistical significance was set at #<0.05 for the treatment effects between the different time points. Table S2. Summary of 23 most up-regulated gene expression for SAL+FOR determined by microarray. Genes involved in exercise and metabolism are marked in bold. [file 40798_2023_630_MOESM1_ESM.docx]

**Supplemental 1**

**Figures and Tables**

**Sports Medicine**

**Title**

**Acute effects of single vs. combinatory inhaled β2-agonists Salbutamol and Formoterol on time trial performance, lung function, metabolic and endocrine variables**

Daniel A. Bizjak, Dorle Nussbaumer, Kay Winkert, Gunnar Treff, Kensuke Takabajashi, Lennart Mentz, Franziska Schober, Jasmine-Lèonike Buhl, Lucas John, Jens Dreyhaupt, Luise Steeb, Lukas C. Harps^,^ Maria K. Parr³, Patrick Diel, Martina Zügel, Jürgen M. Steinacker

**Corresponding author**

Daniel Alexander Bizjak

Department of Internal Medicine, Division of Sports and Rehabilitation Medicine, University Hospital Ulm, 89075 Ulm, Germany

daniel.bizjak@uniklinik-ulm.de

**Table 1:** Schedule illustrating interventions of the ELSA trial.

| **Study arm – Testing days** | | | | | | | | | | |
| --- | --- | --- | --- | --- | --- | --- | --- | --- | --- | --- |
|  | **Day**  **1** | | | | | | | | **Day 2** | **Day 3 or 4** |
| **_Procedures_** | **_Pre_** | **_Study medication (SM)_** | **_10 min after SM_** | **_20 min after_**  **_SM_** | **_15-45 min after_**  **_TT_** | **_1 h after TT_** | **_2 hrs. after TT_** | **_3 hrs. after TT_** | **_24 hrs. after_**  **_TT_** | **_Phone Call_** |
| **_Anthropometry_** | **_X_** |  |  |  |  |  |  |  |  |  |
| **_BP, HR_** | **_X_** |  | **_X_** |  | **_X_** | **_X_** | **_X_** | **_X_** | **_X_** |  |
| **_Blood, urine_** | **_X_** |  |  |  | **_X_** |  |  | **_X_** | **_X_** |  |
| **_Respiratory testing_** | **_X_** |  |  |  | **_X_** |  |  | **_X_** | **_X_** |  |
| **_Inhalation SM_** | **_X_** | **_X_** |  |  |  |  |  |  |  |  |
| **_Time Trial (TT), cardiac output Q̇_** |  |  |  | **_X_** |  |  |  |  |  |  |
| **_ECG_** |  |  |  |  | **_X_** |  |  |  | **_X_** |  |
| **_Echocardiography_** |  |  |  |  | **_X_** |  |  |  |  |  |
| **_Muscle biopsy_** |  |  |  |  |  |  |  | **_X_** |  |  |
| **_Conc. Dis./Med._** | **_X_** |  |  |  |  |  |  |  | **_X_** |  |
| **_Adverse Events_** | **_X_** |  |  |  |  |  |  | **_X_** | **_X_** | **_X_** |

Blood pressure (BP), heart rate (HR), electrocardiogram (ECG), concomitant diseases and/or medications (conc. Dis./Med.).

**Figure 1: β2-Agonist (salbutamol (SAL) / formoterol (FOR) / combination SAL+FOR) detection in urine samples for each study arm (A1-A4) by LC-MS.** The different medication application could be reliable detected and assigned to the respective study arm. An example analysis is given for one participant, where A1 (PLA), A2 (SAL+FOR), A3 (SAL) and A4 (FOR) could be assigned without unblinding.

**Figure 2: Hormonal response of the participants before (Pre) β2-agonist (salbutamol (SAL) / formoterol (FOR) / combination SAL+FOR) application, directly after the time trial (Post) and 3 h after the time trial (3h Post) in blood serum.** The different medication application resulted in a treatment effect for the whole group for (F) follicle-stimulating hormone (FSH), (H) insulin and (J) luteinizing hormone. No treatment effect was observed for (A) adrenaline, (B) noradrenaline, (C) tumour-growth-factor beta (TGF-beta), (D) insulin growth factor 1 (IGF-1), (E) adrenocorticotropic hormone (ACTH), (G) n-terminal prohormone of brain natriuretic peptide (NT-BNP) or (I) cortisol. Statistical significance was set at #<0.05 for the treatment effects between the different time points.

**Table 2: Summary of 23 most up-regulated gene expression for SAL+FOR determined by microarray.** Genes involved in exercise and metabolism are marked in bold.

| **Gene Symbol** | **Description** | **Chromosome** | **Group** | **Start** | **Stop** |
| --- | --- | --- | --- | --- | --- |
| PDE4B | phosphodiesterase 4B, cAMP-specific | chr1 | Multiple_Complex | 65792510 | 66374579 |
| **ATF3** | **activating transcription factor 3** | **chr1** | **Multiple_Complex** | **212565334** | **212620777** |
| TRIM63 | tripartite motif containing 63, E3 ubiquitin protein ligase | chr1 | Multiple_Complex | 26051304 | 26068436 |
| TXNIP | thioredoxin interacting protein | chr1 | Multiple_Complex | 145992435 | 145996631 |
| ACKR3 | atypical chemokine receptor 3 | chr2 | Multiple_Complex | 236558365 | 236582358 |
| **ATP1B3** | **ATPase, Na+/K+ transporting, beta 3 polypeptide** | **chr3** | **Multiple_Complex** | **141876124** | **141926540** |
| FAM134B | family with sequence similarity 134, member B | chr5 | Multiple_Complex | 16473038 | 16617058 |
| ELL2 | elongation factor, RNA polymerase II, 2 | chr5 | Multiple_Complex | 95885098 | 95962071 |
| SLC22A3 | solute carrier family 22 (organic cation transporter), member 3 | chr6 | Multiple_Complex | 160348268 | 160452581 |
| HDAC9 | Jeck2013 ALT_ACCEPTOR, ALT_DONOR, coding, INTERNAL, intronic best transcript NM_178423 | chr7 | NonCoding | 18654763 | 18656987 |
| **PDK4** | **pyruvate dehydrogenase kinase, isozyme 4** | **chr7** | **Multiple_Complex** | **95583497** | **95596613** |
| **LPL** | **lipoprotein lipase** | **chr8** | **Multiple_Complex** | **19901717** | **19967259** |
| KLF10 | Kruppel-like factor 10 | chr8 | Multiple_Complex | 102648775 | 102655902 |
| **CREM** | **cAMP responsive element modulator** | **chr10** | **Multiple_Complex** | **35126791** | **35212958** |
| SIK2 | salt-inducible kinase 2 | chr11 | Multiple_Complex | 111602391 | 111730853 |
| E2F8 | E2F transcription factor 8 | chr11 | Multiple_Complex | 19224063 | 19241655 |
| PPP2R1B | protein phosphatase 2, regulatory subunit A, beta | chr11 | Multiple_Complex | 111726908 | 111766445 |
| SIX1 | SIX homeobox 1 | chr14 | Multiple_Complex | 60638599 | 60658259 |
| PMP22 | peripheral myelin protein 22 | chr17 | Multiple_Complex | 15229777 | 15265357 |
| CHMP1B | charged multivesicular body protein 1B | chr18 | Multiple_Complex | 11851390 | 11854449 |
| ZNF331 | zinc finger protein 331 | chr19 | Multiple_Complex | 53520923 | 53580269 |
| SIK1 | salt-inducible kinase 1 | chr21 | Multiple_Complex | 43414515 | 43427128 |
| DGKD | diacylglycerol kinase, delta 130kDa |  | NonCoding |  |  |
